# Significance of maintenance therapy after HDT/ASCT in symptomatic multiple myeloma: A multicenter retrospective analysis in Kansai Myeloma Forum

**DOI:** 10.1002/jha2.284

**Published:** 2021-10-20

**Authors:** Aya Nakaya, Hirohiko Shibayama, Eiji Nakatani, Yuji Shimura, Satoru Kosugi, Hirokazu Tanaka, Shin‐Ichi Fuchida, Junya Kanda, Nobuhiko Uoshima, Hitomi Kaneko, Kazunori Imada, Kensuke Ohta, Tomoki Ito, Hideo Yagi, Satoshi Yoshihara, Masayuki Hino, Chihiro Shimazaki, Akifumi Takaori‐Kondo, Junya Kuroda, Itaru Matsumura, Yuzuru Kanakura, Shosaku Nomura

**Affiliations:** ^1^ First Department of Internal Medicine Kansai Medical University Osaka Japan; ^2^ Department of Hematology and Oncology Osaka University Graduate School of Medicine Osaka Japan; ^3^ Graduate School of Public Health Shizuoka Graduate University of Public Health Shizuoka Japan; ^4^ Division of Hematology and Oncology, Department of Medicine Kyoto Prefectural University of Medicine Kyoto Japan; ^5^ Department of Internal Medicine (Hematology) Toyonaka Municipal Hospital Osaka Japan; ^6^ Department of Hematology and Rheumatology Kindai University Faculty of Medicine Osaka Japan; ^7^ Department of Hematology Japan Community Health Care Organization Kyoto Kuramaguchi Medical Center Kyoto Japan; ^8^ Department of Hematology and Oncology, Graduate School of Medicine Kyoto University Kyoto Japan; ^9^ Department of Hematology Japanese Red Cross Kyoto Daini Hospital Kyoto Japan; ^10^ Department of Hematology Japanese Red Cross Osaka Hospital Osaka Japan; ^11^ Hematology Ohta Clinic Shinsaibashi Osaka Japan; ^12^ Department of Hematology and Oncology Nara Prefecture General Medical Center Nara Japan; ^13^ Division of Hematology, Department of Internal Medicine Hyogo College of Medicine Hyogo Japan; ^14^ Department of Hematology Osaka City University Graduate School of Medicine Osaka Japan; ^15^ Department of Hematology Sumitomo Hospital Osaka Japan; ^16^ Kansai Myeloma Forum Osaka Japan

**Keywords:** autologous stem cell transplantation, lenalidomide, maintenance therapy, retrospective, symptomatic multiple myeloma

## Abstract

A total of 129 symptomatic patients with multiple myeloma (MM) who underwent high‐dose chemotherapy with autologous stem cell transplantation (HDT/ASCT) were analyzed. The 4‐year overall survival (OS) of patients with maintenance (*n* = 82) was 80%, whereas that of patients without maintenance (*n* = 47) was 72% (*p* = 0.426). The 4‐year progression‐free survival (PFS) of patients with maintenance was 38%, whereas that of patients without maintenance was 27% (*p* = 0.088). Multivariate analysis revealed that an International Staging System score ≥2 was associated with worse PFS (hazard ratio 1.62, *p* = 0.043). Among the 129 patients, two were excluded owing to early relapse, 50 patients achieved complete response (CR), and 77 patients failed to achieve CR. Patients who achieved CR showed better 4‐year PFS than those who failed to achieve CR (41% vs. 30%, *p* = 0.027); however, 4‐year OS was not different (76% vs. 82%, *p* = 0.971). In patients who achieved CR, 4‐year OS with/without maintenance was 74%/81% (*p* = 0.357), 4‐year PFS with/without maintenance was 42%/40% (*p* = 0.954). In patients who failed to achieve CR, the 4‐year OS with/without maintenance was 97%/91% (*p* = 0.107), and 4‐year PFS with/without maintenance was 36%/16% (*p* < 0.001). In patients who failed to achieve CR, maintenance significantly improved the PFS. Maintenance after HDT/ASCT can prolong PFS in patients who fail to achieve CR in real‐world settings.

## 1 INTRODUCTION

High‐dose chemotherapy with autologous stem cell transplantation (HDT/ASCT) has been a standard therapy for newly diagnosed symptomatic patients with multiple myeloma (MM) aged < 65 years. This approach significantly improves outcomes; however, most patients experience disease progression. Therefore, continuous therapies such as consolidation and maintenance have been developed to maintain long‐term disease control. Multiple randomized studies have demonstrated the efficacy of continuous therapies.

Continuous thalidomide therapy has mostly been studied in large studies. These studies showed prolonged progression free survival (PFS)/overall survival (OS) [1]^–^[3]. Long‐term use of thalidomide is also associated with significant neuropathy, which limits its use in maintenance therapy [4, 5].

Lenalidomide has also been studied for maintenance therapy and is currently a standard agent. Three large trials, CALGB 100104 [6], IFM 2005–02 [7], and GIMEMA RV‐209 [8], have evaluated the role of lenalidomide in maintenance therapy. All studies revealed a significant improvement in the PFS. Only CALGB100104 revealed an improvement in OS, while the other two studies failed to reveal it. Meta‐analysis of these three studies estimated that both OS and PFS were prolonged [9]. Myeloma XI is the latest and largest study of lenalidomide, which revealed an improvement in PFS [10]. This study confirmed that lenalidomide maintenance improved PFS in high‐risk patients. All studies demonstrated common adverse events, including neutropenia and an increased risk of secondary malignancy.

Bortezomib has fewer randomized controlled trials (RCTs) on maintenance therapy. The HOVON‐65/GMMG‐HD4 trial is the first study to be referenced [11]. The induction therapies were bortezomib‐based regimens and maintenance therapies were either thalidomide or bortezomib. Patients with bortezomib maintenance showed a better response than those with thalidomide. Although this is not an accurate head‐to‐head comparison, the results support the efficacy of bortezomib as maintenance therapy, suggesting that bortezomib might be beneficial in high‐risk group [12]. GEM05MENOS65 was a phase III trial that compared the combination of thalidomide and bortezomib, thalidomide monotherapy, and interferon. Combination therapy showed longer PFS, but not OS [13].

Owing to these previous studies, maintenance has recently been considered standard care. Several guidelines outside Japan recommend maintenance therapies [14]^–^[16]. However, the optimal agent, dose, combination, and duration have not yet been established. In Japan, it has been clinically acceptable, although the Japanese guidelines describe that maintenance should be performed within trials [17]. Therefore, maintenance was performed using various methods. We retrospectively analyzed the significance of maintenance therapy in real‐world patients with symptomatic MM registered with the Kansai Myeloma Forum (KMF), a study group for plasma cell dyscrasias.

## 2 PATIENTS AND METHODS

The KMF, a study group comprising 73 facilities in the Kansai region of Japan, was established in 2012 to register clinical data of patients with all types of plasma cell dyscrasias to retrospectively analyze treatment strategies and their outcomes. By December 2017, KMF registered the clinical data of 2764 patients with plasma cell dyscrasias, of whom 129 had received HDT/ASCT for symptomatic MM between June 2012 and November 2017.

All patients received bortezomib‐based regimens as induction therapy. All transplantations were initial ones. Maintenance therapy is defined as long‐term continuous therapy initiated within 6 months after HDT/ASCT or therapy, which was intentionally administered by the attending physician as maintenance therapy. This study was conducted as per the ethical principles of the Declaration of Helsinki and was approved by the institutional review boards of all institutions participating in the KMF.

## 3 STATISTICAL ANALYSIS

Continuous and categorical variables were summarized as median (range) and percent, respectively. In two‐group comparisons, Fisher's exact test and Wilcoxon's rank‐sum test were used for continuous and categorical variables, respectively. OS was calculated as the period from the date of ASCT to the event of interest. PFS was defined as the period from the date of ASCT to disease progression or death due to any cause. In the subgroup analysis, patients were divided into two groups according to their response after HDT/ASCT. To avoid overestimation, landmark analysis was adopted. The landmark was put on 3 months after the ASCT. The OS/PFS was calculated from the landmark point to the event of interest, adjusted gender (men), M protein, and performance status (PS). Survival curves were created using the Kaplan–Meier method, and differences were evaluated using the log‐rank test. Univariate and multivariate analyses were performed using the Cox proportional hazards model, and hazard ratios, 95% confidence intervals, and *p* values were calculated. Variables that were significant in univariate regression analysis, existing prognostic factors such as age (> 60 years vs. < 60 years), gender (male vs. female), International Staging System (ISS) (≥2 vs. < 2), PS (≥2 vs. < 2), IgG M protein, and maintenance presence or absence were used as explanatory variables in the multivariate model. All statistical tests were two sided, and statistical significance was set at *p *< 0.05. The 95% confidence interval for the annual OS/PFS and rates was calculated using Greenwood's formula. All statistical analyses were performed using EZR (Saitama Medical Center, Jichi Medical University, Saitama, Japan), which is a graphical user interface for R version 2.13.0 (R Foundation, Vienna, Austria) [18]. More precisely, a modified version of R Commander (version 1.6‐3) was designed to incorporate statistical functions frequently used in biostatistics.

## 4 RESULTS

### 4.1 Patients’ characteristics

From June 2012 to November 2017, 129 patients who underwent HDT/ASCT with symptomatic MM were registered in the KMF database. Of the 129 patients, 82 received maintenance therapy. The median observational period was 3.9 years (range, 0.3–8.4 years). The clinical characteristics of the study patients are summarized in Table 1. The median age of patients with maintenance was 58 years (range, 36–74 years), and that of patients without maintenance was 59 years (range, 31–72 years). Among all the patients, IgG M protein was the most frequent. However, among patients without maintenance, 32% of them had IgA, whereas 11% of patients with maintenance had IgA (*p* = 0.002).

**TABLE 1:** Characteristics of patients with/without maintenance

Variable [reference]	Category or statistics	Maintenance (*n* = 82)	Without maintenance (*n* = 47)	*p*‐value
Age (y/o)	Median (range)	58 (36–74)	59 (31–72)	0.772
Gender (%) [Women]	Men	55	57	0.887
ISS (%)	I	40	44	0.054
	II	43	28	
	III	17	28	
PS (%) [< 2]	≥2	15	13	0.839
M protein (%)	IgG	65	55	<0.001
	IgA	11	32	
	BJP	23	13	
	IgD	1	0	
Majority of free light chain (%) [λ]	κ	68	62	0.449
Response after ASCT (%) [< CR]	≥CR	32	53	0.039
Response after ASCT (%) [< VGPR]	≥VGPR	80	87	0.486
Observational period (y)	Median (range)	3.9 (0.3–8.4)	3.8 (0.3–7.1)	1.000

*Note*: IgG M protein is the most frequent in both groups. However, in patients without maintenance, IgA M protein is observed more frequently than in those with maintenance (32% vs. 11%). Approximately 32% of patients with maintenance and 53% of patients without maintenance have achieved CR after ASCT. Abbreviations: ASCT, autologous stem cell transplantation; BJP, Bence Jones protein; CR, complete response; ISS, International Staging System; PS, performance status; VGPR, very good partial response.

### 4.2 Maintenance regimens

Eighty‐two patients received maintenance therapy. Among them, 76% (*n* = 62) received immunomodulator‐based regimens. Of these, 33 patients received low‐dose lenalidomide (5–15 mg) plus dexamethasone, 26 received low‐dose lenalidomide monotherapy, and three patients received thalidomide. There were 24% patients who received proteasome inhibitor (PI)‐based regimens. Two patients received bortezomib triplet regimens, 14 patients received bortezomib plus dexamethasone, and four received bortezomib monotherapy. Among the 82 patients, seven were ongoing, while 75 patients discontinued treatment. The most frequent reason for discontinuation was disease progression (*n* = 37; 46%), followed by CR (*n* = 15; 18%), adverse events (*n* = 11; 13%), patient refusal (*n* = 4; 5%), and treatment plan (*n* = 4; 5%). Among patients who had adverse events (*n* = 11), three had a severe infection, three had fatigue, one patient had peripheral neuropathy, one had a skin rash, one had secondary malignancy (lung cancer), and two patients were unknown.

### 4.3 Outcome

**FIGURE 1:**
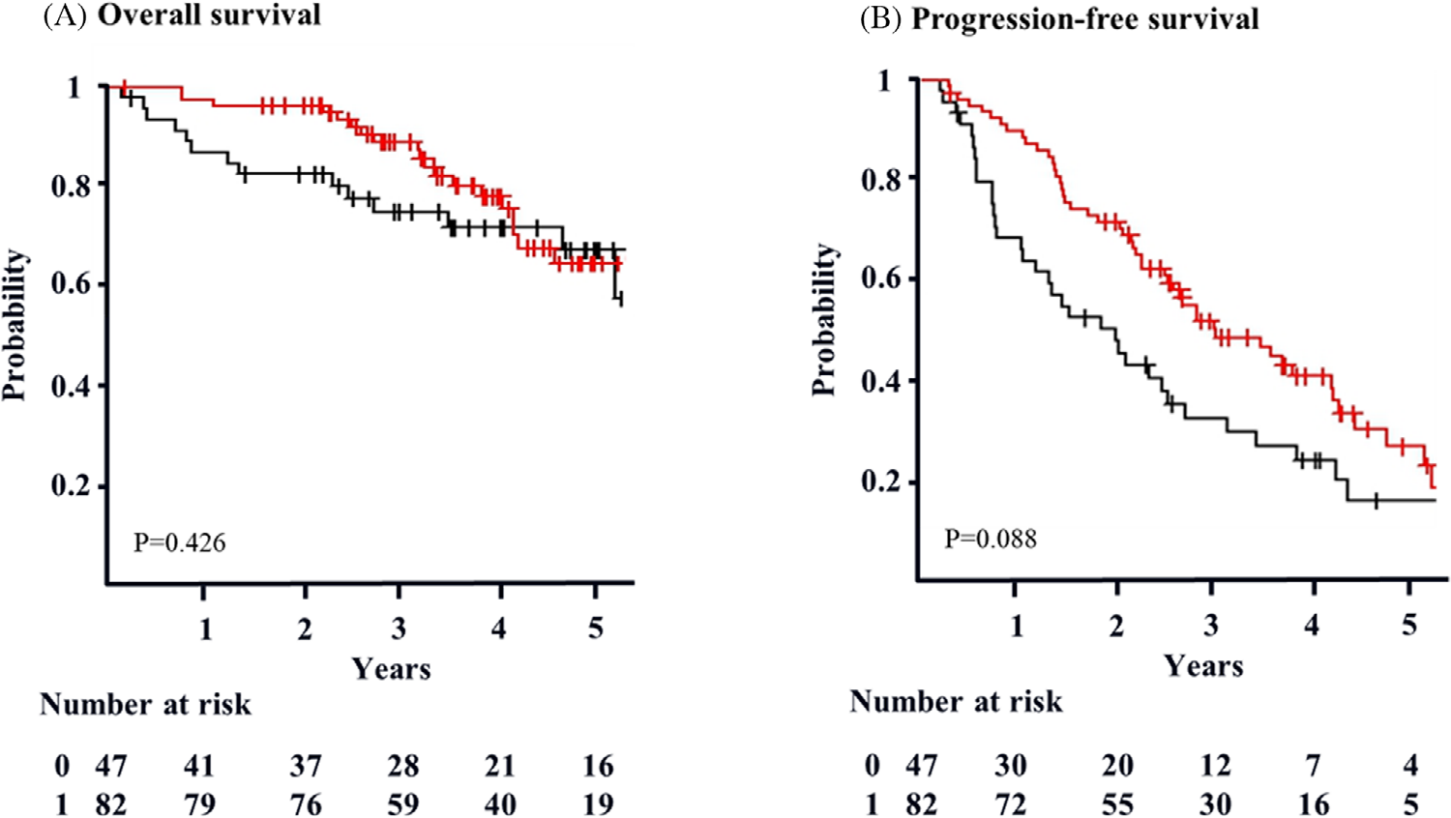
Overall survival and progression‐free survival. (A) The 4‐year OS of patients with maintenance (red) is 80% (95% CI: 68–88%). The 4‐year OS of patients without maintenance (black) is 72% (95% CI: 56–83%) (*p* = 0.426). Survival is calculated from the transplant day. (B) The 4‐year PFS with maintenance (red) is 38% (95% CI: 26–50%). The 4‐year PFS without maintenance (black) is 27% (95% CI: 15–41%) (*p* = 0.088). Survival is calculated from the transplant day

The 4‐year OS of patients with maintenance was 80% (95% CI: 68–88%), and that of patients without maintenance was 72% (95% CI: 56–83%) (*p *= 0.426) (Figure 1A). The 4‐year PFS of patients with maintenance was 38% (95% CI: 26–50%), and that of patients without maintenance was 27% (95% CI: 15–41%) (*p *= 0.088) (Figure 1B). Approximately 32% of patients with maintenance and 53% without maintenance achieved CR after HDT/ASCT (*p *= 0.030). The median duration of maintenance for patients who achieved CR was 23 months. Concerning secondary malignancy, a patient with maintenance therapy developed lung cancer, whereas another without maintenance therapy developed pancreatic cancer.

### 4.4 Prognostic factors

The results of univariate analysis were showed in Supplementary Table 1. Multivariate analysis was performed to identify risk factors (Table 2). An ISS ≥ 2 was an independent predictor of PFS (hazard ratio 1.62, 95% CI: 1.02–2.59, *p *= 0.043).

**TABLE 2:** Result of multivariate Cox regression analysis for OS and PFS

Variable	Category	Overall survival			Progression‐free survival		
		Hazard ratio	95% CI	*p*‐value	Hazard ratio	95% CI	*p*‐value
Age (y/o)	≥60	1.46	0.74‐2.86	0.277	1.07	0.69‐1.67	0.765
Gender	Men	1.81	0.89‐3.68	0.101	1.27	0.83‐1.96	0.275
ISS	≥2	1.48	0.75‐2.91	0.264	1.62	1.02‐2.59	0.043
PS	≥2	0.29	0.08‐1.01	0.052	0.80	0.43‐1.48	0.474
M protein	IgG	0.55	0.28‐1.10	0.090	0.83	0.52‐1.31	0.415
Maintenance	+	0.92	0.46‐1.82	0.807	0.70	0.45‐1.08	0.106

*Note*: An ISS ≥2 predicted worse PFS, while other factors failed to predict OS and PFS. Abbreviations: CI, confidence interval; ISS, International Staging System; OS, overall survival; PFS, progression‐free survival; PS, performance status.

### 4.5 Subgroup analysis

#### 4.5.1 Characteristics and landmark analysis for the patients with/without CR

In patients without maintenance, 53% of patients without maintenance therapy achieved CR after HDT/ASCT. We assumed that the response after HDT/ASCT may have influenced the efficacy of maintenance therapy. Therefore, we divided the patients into two groups: patients who achieved CR (*n* = 50) and those who failed to achieve CR (*n* = 77). We placed the landmark point of 3 months after ASCT to avoid selection bias. Two patients were excluded owing to early relapse. A total of 127 patients were included in the analysis.

The clinical characteristics of the patients are shown in Table 3. There were 24% of patients with CR who had a PS score ≥2, whereas it was 8% in patients without CR (*p *= 0.003). Seventy percent of patients without CR had IgG, and 46% of patients with CR had IgG. On the contrary, 30% of patients with CR had IgA, and 12% of patients without CR had IgA (*p *< 0.001).

**TABLE 3:** Characteristics of patients who achieved CR and failed to achieve CR

Variable [reference]	Category or statistics	≥CR (*n* = 50)	<CR (*n* = 77)	*p*‐value
Age (y/o)	Median (range)	60 (36–74)	58 (31–74)	0.879
Gender (%) [Women]	Men	44	64	0.007
ISS (%)	I	46	40	0.666
	II	36	38	
	III	18	22	
PS (%) [< 2]	≥2	24	8	0.003
M protein (%)	IgG	46	70	<0.001
	IgA	30	12	
	BJP	24	17	
	IgD	0	1	
Majority of Free light chain (%) [λ]	κ	66	65	1.000
Observational period (y)	Median (range)	4.0 (0.4–6.7)	3.9 (0.3–8.4)	0.141

*Note*: Approximately 24% of the patients achieved CR with a PS ≥2. IgG M protein is the most frequent in both groups. IgG M protein is observed more frequently in patients who failed to achieve CR, whereas IgA protein is seen more frequently in those who achieved CR.

Abbreviations: BJP, Bence Jones protein; CR, complete response; ISS, International Staging System; PS, performance status.

**FIGURE 2:**
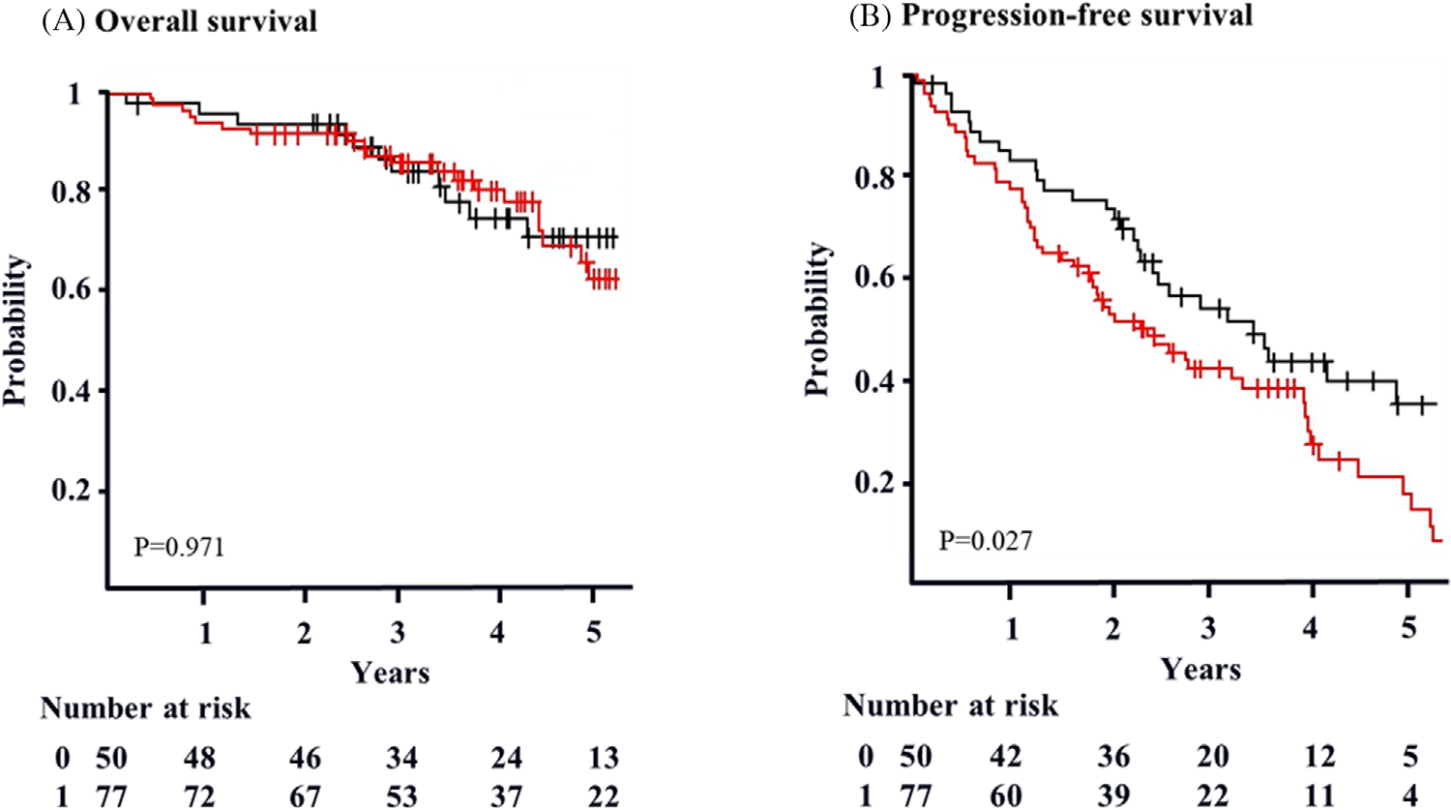
Overall survival and progression‐free survival according to response after ASCT, adjusted gender [men], PS, and M protein (landmark analysis). (A) The 4‐year OS of patients who achieved CR (black) is 76% (95% CI: 64–91%). The 4‐year OS of patients who failed to achieve CR (red) is 82% (95% CI: 73–91%) (*p* = 0.971). Survival is calculated from 3 months after ASCT (landmark point). (B) The 4‐year PFS of patients who achieved CR (black) is 41% (95% CI: 29–60%). The 4‐year PFS of patients who failed to achieve CR (red) is 30% (95% CI: 20–46) (*p* = 0.027)

**FIGURE 3:**
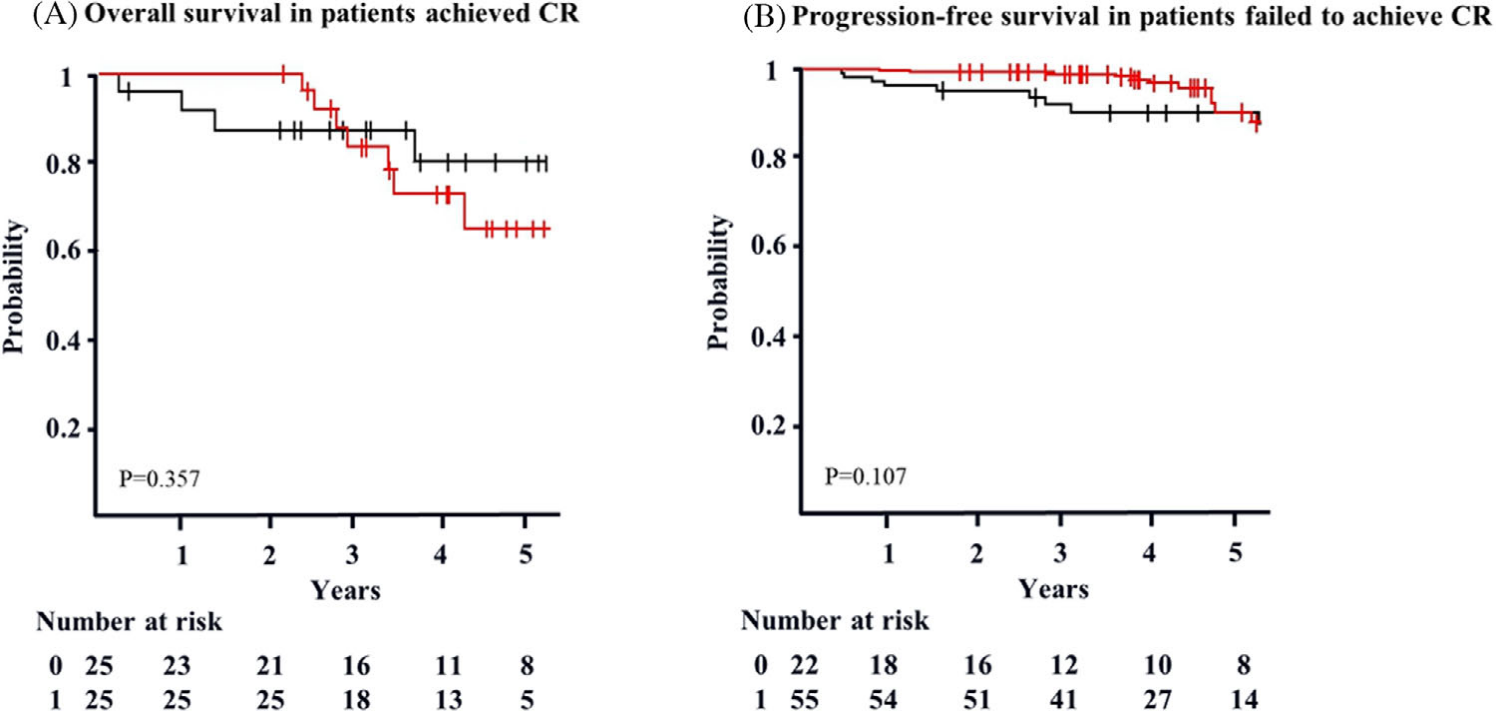
Overall survival according to response in patients with/without maintenance, adjusted gender [men], PS,, and M protein (landmark analysis). a. The 4‐year OS of patients who achieved CR with maintenance (red) is 74% (95% CI: 56–97%). The 4‐year OS of patients without maintenance (black) is 81% (95% CI: 65–100%) (*p* = 0.357). b. The 4‐year OS of patients who failed to achieve CR with maintenance (red) is 97% (95% CI: < 1–100%). The 4‐year OS of patients without maintenance (black) is 91% (95% CI: < 1–100%) (p = 0.107)

**FIGURE 4:**
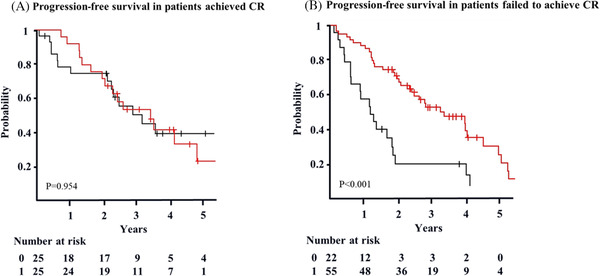
PFS according to response in patients with/without maintenance, adjusted gender [Men], PS, and M protein (landmark analysis). a. The 4‐year PFS of patients who achieved CR with maintenance (red) is 42% (95% CI: 25–70%). The 4‐year PFS of patients without maintenance (black) is 40% (95% CI: 23–69%) (*p* = 0.954). (B) The 4‐year PFS of patients who failed to achieve CR with maintenance (red) is 36% (95% CI: 24–57%). The 4‐year PFS of patients without maintenance (black) is 16% (95% CI: 6–45%) (*p* < 0.001)

The OS/PFS was calculated from the landmark point to the event of interest, adjusted gender (men), M protein, and PS. The 4‐year OS was not different between patients who achieved CR and those who did not (76% vs. 82%, *p *= 0.971) (Figure 2A), whereas 4‐year PFS revealed a significant difference (41% vs. 30%, *p *= 0.027) (Figure 2B). To evaluate the efficacy of maintenance therapy, we analyzed the 4‐year OS and PFS of patients who achieved CR and those who did not. The 4‐year OS of patients with CR was 74% (95% CI: 56–97%) with maintenance therapy and 81% (95% CI: 65–100%) without maintenance therapy (*p *= 0.357) (Figure 3A). The 4‐year OS of patients without CR was 97% (95% CI: < 1–100%) with maintenance therapy and 91% (95% CI: < 1–100%) without maintenance therapy (*p *= 0.107) (Figure 3B). The 4‐year PFS of patients with CR was 42% (95% CI: 25–70%) with maintenance therapy and 40% (95% CI: 23–69%) without maintenance therapy (*p *= 0.954) (Figure 4A). The 4‐year PFS of patients without CR was 36% (95% CI: 24–57%) with maintenance therapy and 16% (95% CI: 6–45%) without maintenance therapy (*p *< 0.001) (Figure 4B).

## 5 DISCUSSION

Although our results failed to show the efficacy of maintenance therapy after HDT/ASCT, it reflected the outcome in a real‐world setting. Moreover, in patients without maintenance, 53% of them had achieved CR after HDT/ASCT. This indicates that the physician in charge might feel that it is not necessary to administer maintenance therapy to patients who have achieved CR after HDT/ASCT. Our subgroup analysis supports this hypothesis. In our subgroup analysis, patients were divided into two groups: those who achieved CR or those who failed to achieve CR. The latter group showed a statistically significant improvement in PFS with maintenance therapy.

The notable point of our study is that we have patients who discontinued maintenance as they achieved CR. In RCTs, maintenance continues until disease progression occurs. As the purpose of maintenance is clearer in a real‐world setting, physicians discontinued maintenance when the goal was achieved. Among the patients who stopped maintenance therapy (*n* = 15), seven relapsed after discontinuing maintenance while eight remained in CR. Owing to the small number of cases, we cannot conclude the pros and cons to discontinue maintenance.

Another important finding suggests a challenge that should be addressed in the near future. During the period of this study, several novel stronger agents were developed. We included 83 patients who relapsed after HDT/ASCT (53 patients with maintenance and 30 without maintenance). Twelve percent of them were treated using carfilzomib (*n* = 10), 12% received ixazomib (n = 10), and 9% received daratumumab (*n* = 7) as salvage therapy. The use of new novel agents as salvage therapy for relapse after HDT/ASCT might have a strong impact on OS in the current study. This might be one reason why we failed to reveal a difference in 4‐year OS. We tried to analyze survival from relapse after maintenance; however, the number of patients was too small and the observation period was too short. Therefore, we shall analyze it when more cases are accumulated in the future.

Moreover, all patients who received ixazomib were switched from lenalidomide maintenance. It must be easy to change oral agents from oral agents in outpatient services. Ixazomib has been studied as a maintenance therapy, and its efficacy has been confirmed [19]. However, it has not been officially approved for use in maintenance during the study period. It may become an alternative agent for maintenance therapy owing to its convenience as an oral agent and its mechanism of action as a PI.

Another question arises: Is maintenance therapy necessary in the presence of new novel agents? It might not be too late to start new novel agents after relapse. We will have to evaluate the necessity of maintenance therapy in the new novel agent era.

The most frequent reason for discontinuation was disease progression following adverse events. The frequency of adverse events was similar to that reported in previous reports. Secondary malignancies were observed in only two patients. A patient with maintenance developed lung cancer, while another without maintenance developed pancreatic cancer. As most of our patients received alkylating free induction (bortezomib‐based regimens), this might account for this small preference for secondary malignancy.

Our study had several limitations. First, this was a retrospective study, and relatively few patients were analyzed. Second, owing to the registry, detailed information, such as surface markers and chromosomal abnormalities, was difficult to collect. Therefore, we were unable to identify high‐risk patients. Third, we defined the maintenance therapy which was started within 6 months after HDT/ASCT. There must be an immortal bias. However, actual number of the patients who died during the period was three. Therefore, we think the immortal bias hardly influence the result. Fourth, it has been reported that a minimal residual disease (MRD) is important to predict outcomes [20]; however, multiparameter flow cytometry has only been approved in Japan; therefore, our patients lacked these data. Owing to the limitation of MRD information, the CR was the only surrogate marker of the residual amount of the myeloma clone in this study. In the future, MRD may become a decision‐making marker for maintenance after HDT/ASCT.

Here, we report the evaluation of maintenance therapy after HDT/ASCT for transplant‐eligible MM patients in a real‐world setting. Although our results failed to show the efficacy of maintenance of OS and PFS, the subgroup analysis showed that patients who failed to achieve CR improved PFS with maintenance therapy. Our results indicate that physicians’ thinking patterns and outcomes of maintenance in the real world. Further investigations are required to assess the optimal strategy of maintenance, the influence of MRD, and posttreatment for relapse after HDT/ASCT.

## CONFLICT OF INTEREST

This work was supported by Bristol‐Myers Squibb/Celgene K.K.

All authors received research funding from Ono Pharmaceutical Co., Ltd., Fujimoto Pharmaceutical Corporation. HT, HS, AT, IM, JKA, TI, KI, SK, and SY received honoraria from Novartis Pharma K.K. JKU, HT, JKA, AT, HS, IM, SF, TI, KI, MH, SK, and SY received honoraria from Bristol‐Myers Squibb/Celgene K.K. IM, HS, JKU, SF, MH, and SY received honoraria from Janssen Pharmaceutical K.K. SF, JKA, TI, HS, KI, MH, and SK received honoraria from Takeda Pharmaceutical Company, Limited. AT, HS, JKA, KI, MH, and SK received honoraria from Kyowa Kirin Co., Ltd. HS, JKA, KI, MH, and SK received honoraria from Chugai Pharmaceutical Co., Ltd. At, JKA, IM, KI, MH, and SK received honoraria from Astellas Pharma Inc. SF, HS, MH, and SY received honoraria from Sanofi K.K. SF, HS, MH, SK, and SY received honoraria from Ono Pharmaceutical Co., Ltd. JKA, IM, KI, and MH received honoraria from Otsuka Pharmaceutical Co., Ltd. JKA and IM received honoraria from Daiichi Sankyo Company, Limited. IM received honoraria from Pfizer Japan Inc. and Amgen inc. IM and TI received honoraria from AbbVie GK. AT, JKA, and MH received honoraria from MSD K.K. HS received honoraria from AstraZeneca K.K., Sanofi K.K. AT, KI, and MH received honoraria from Nippon Shinyaku Co., Ltd. JKA received honoraria from JCR Pharmaceuticals Co., Ltd. JKU and SK received honoraria from Fujimoto Pharmaceutical Corporation. KI and MH received honoraria from Dainippon Sumitomo Pharma Co., Ltd. MH received honoraria from Pfizer Japan Inc., Japan Blood Products Organization, Mochida Pharmaceutical CO., Ltd., and Mundipharma K.K. MH received honoraria from Eisai Co., Ltd. JKU, AT, HS, and TI received research funding from Bristol‐Myers Squibb. IM, TI, and HS received research funding from AbbVie GK. AT and HS received research funding from Ono Pharmaceutical Co., Ltd. IM, HS, and SY received research funding from Chugai Pharmaceutical Co., Ltd. IM and HS received research funding from Novartis Pharma K.K. IM received research funding from Takeda Pharmaceutical Company, Limited., Pfizer Japan Inc., Eisai Co., Ltd., and Alexion Pharmaceuticals, Inc. HS received research funding from Janssen Pharmaceutical K.K., Sanofi K.K., and AstraZeneca K.K. MH received research funding from Taiho Pharmaceutical Co., Ltd., Teijin Limited, Eisai Co., Ltd., Nihon Pharmaceutical Co., Ltd., and JCR Pharmaceuticals Co., Ltd. AT, IM received scholarship grant from Takeda Pharmaceutical Company, Limited., Chugai Pharmaceutical Co., Ltd., Eisai Co., Ltd., Nippon Shinyaku Co., Ltd., Astellas Pharma Inc., Otsuka Pharmaceutical Co., Ltd., Sanofi K.K., Shionogi & Co., Ltd., and AbbVie GK. AT, HT, and IM received scholarship grant from Kyowa Kirin Co., Ltd. AT received scholarship grant from Ohara Pharmaceutical Co., Ltd, and Kinshikouraininjin., the Japanese Society of Hematology. IM received scholarship grant from Ono Pharmaceutical Co., Ltd., Dainippon Sumitomo Pharma Co., Ltd., Asahi Kasei Pharma Corporation., Taiho Pharmaceutical Co., Ltd., Mitsubishi Tanabe Pharma Corporation., Novartis Pharma K.K., and MSD K.K. JKU received scholarship grant from Fujimoto Pharmaceutical Corporation.

## AUTHORS' CONTRIBUTIONS

AN and HS designed the study. AN and EN performed the statistical analyses, interpreted the data. AN drafted the manuscript. HY, HK, SK, TK, YA, TK, YK, SF, NU, EK, HU, YS, TT, FU, KO, TH, KM, MK, MS, HT, CS, MH, JK, ATK, SN, and IM provided patient data. All coauthors approved the final version of the manuscript.

## Supporting information



Table S1

## References

[1] Attal M , Harousseau JL , Leyvraz S , Doyen C , Hulin C , Benboubker L , et al. Maintenance therapy with thalidomide improves survival in patients with multiple myeloma. Blood. 2006;108(10):3289‐94. 10.1182/blood-2006-05-022962 16873668

[2] Barlogie B , Pineda‐Roman M , van Rhee F , Haessler J , Anaissie E , Hollmig K , et al. Thalidomide arm of total therapy 2 improves complete remission duration and survival in myeloma patients with metaphase cytogenetic abnormalities. Blood. 2008;112(8):3115‐21. 10.1182/blood-2008-03-145235 18492953 PMC2569166

[3] Spencer A , Prince HM , Roberts AW , Prosser IW , Bradstock KF , Coyle L , et al. Consolidation therapy with low‐dose thalidomide and prednisolone prolongs the survival of multiple myeloma patients undergoing a single autologous stem‐cell transplantation procedure. J Clin Oncol. 2009;27(11):1788‐93. 10.1200/JCO.2008.18.8573 19273705

[4] Lokhorst HM , van der Holt B , Zweegman S , Vellenga E , Croockewit S , van Oers MH , et al. A randomized phase 3 study on the effect of thalidomide combined with adriamycin, dexamethasone, and high‐dose melphalan, followed by thalidomide maintenance in patients with multiple myeloma. Blood. 2010;115(6):1113‐20. 10.1182/blood-2009-05-222539 19880501

[5] Morgan GJ , Gregory WM , Davies FE , Bell SE , Szubert AJ , Brown JM , et al. The role of maintenance thalidomide therapy in multiple myeloma: MRC myeloma IX results and meta‐analysis. Blood. 2012;119(1):7‐15. 10.1182/blood-2011-06-357038 22021371

[6] McCarthy PL , Owzar K , Hofmeister CC , Hurd DD , Hassoun H , Richardson PG , et al. Lenalidomide after stem‐cell transplantation for multiple myeloma. N Engl J Med. 2012;366(19):1770‐81. 10.1056/NEJMoa1114083 22571201 PMC3744390

[7] Attal M , Lauwers‐Cances V , Marit G , Caillot D , Moreau P , Facon T , et al. Lenalidomide maintenance after stem‐cell transplantation for multiple myeloma. N Engl J Med. 2012;366(19):1782‐91. 10.1056/NEJMoa1114138 22571202

[8] Palumbo A , Cavallo F , Gay F , Di Raimondo F , Ben Yehuda D , Petrucci MT , et al. Autologous transplantation and maintenance therapy in multiple myeloma. N Engl J Med. 2014;371(10):895‐905. 10.1056/NEJMoa1402888 25184862

[9] McCarthy PL , Holstein SA , Petrucci MT , Richardson PG , Hulin C , Tosi P , et al. Lenalidomide maintenance after autologous stem‐cell transplantation in newly diagnosed multiple myeloma: a meta‐analysis. J Clin Oncol. 2017;35(29):3279‐3289. 10.1200/JCO.2017.72.6679 28742454 PMC5652871

[10] Jackson GH , Davies FE , Pawlyn C , Cairns DA , Striha A , Collett C , et al. Lenalidomide maintenance versus observation for patients with newly diagnosed multiple myeloma (Myeloma XI): a multicentre, open‐label, randomised, phase 3 trial. Lancet Oncol. 2019;20(1):57‐73. 10.1016/S1470-2045(18)30687-9 30559051 PMC6318225

[11] Sonneveld P , Schmidt‐Wolf IG , van der Holt B , El Jarari L , Bertsch U , Salwender H , et al. Bortezomib induction and maintenance treatment in patients with newly diagnosed multiple myeloma: results of the randomized phase III HOVON‐65/GMMG‐HD4 trial. J Clin Oncol. 2012;30(24):2946‐55. 10.1200/JCO.2011.39.6820 22802322

[12] Neben K , Lokhorst HM , Jauch A , Bertsch U , Hielscher T , van der Holt B , et al. Administration of bortezomib before and after autologous stem cell transplantation improves outcome in multiple myeloma patients with deletion 17p. Blood. 2012;119(4):940‐8. 10.1182/blood-2011-09-379164 22160383

[13] Rosiñol L , Oriol A , Teruel AI , de la Guía AL , Blanchard M , de la Rubia J , et al. Bortezomib and thalidomide maintenance after stem cell transplantation for multiple myeloma: a PETHEMA/GEM trial. Leukemia. 2017;31(9):1922‐1927. 10.1038/leu.2017.35 28111466

[14] Dimopoulos MA , Moreau P , Terpos E , Mateos MV , Zweegman S , Cook G , et al. Multiple myeloma: EHA‐ESMO Clinical Practice Guidelines for diagnosis, treatment and follow‐up†. Ann Oncol. 2021;32(3):309‐322. 10.1016/j.annonc.2020.11.014 33549387

[15] Gay F , Engelhardt M , Terpos E , Wäsch R , Giaccone L , Auner HW , et al. From transplant to novel cellular therapies in multiple myeloma: European Myeloma Network guidelines and future perspectives. Haematologica. 2018;103(2):197‐211. 10.3324/haematol.2017.174573 29217780 PMC5792264

[16] National Comprehensive Cancer Network . Multiple Myeloma (version 4.2021). https://www.nccn.org/professionals/physician_gls/PDF/myeloma.pdf Accessed 1 March 2021 (in Japanese).

[17] JSH guideline for Multiple Myeloma. http://www.jshem.or.jp/gui‐hemali/table.html. Accessed March 1, 2021.

[18] Kanda Y . Investigation of the freely available easy‐to‐use software 'EZR' for medical statistics. Bone Marrow Transplant. 2013;48(3):452‐8. 10.1038/bmt.2012.244 23208313 PMC3590441

[19] Dimopoulos MA , Gay F , Schjesvold F , Beksac M , Hajek R , Weisel KC , et al. Oral ixazomib maintenance following autologous stem cell transplantation (TOURMALINE‐MM3): a double‐blind, randomised, placebo‐controlled phase 3 trial. Lancet. 2019;393(10168):253‐264. 10.1016/S0140-6736(18)33003-4 30545780

[20] Munshi NC , Avet‐Loiseau H , Rawstron AC , Owen RG , Child JA , Thakurta A , et al. Association of minimal residual disease with superior survival outcomes in patients with multiple myeloma: a meta‐analysis. JAMA Oncol. 2017;3(1):28‐35. 10.1001/jamaoncol.2016.3160 27632282 PMC5943640

