# Supplementary material for: Significance of maintenance therapy after HDT/ASCT in symptomatic multiple myeloma: A multicenter retrospective analysis in Kansai Myeloma Forum
Source: EJHaem. 2021 Oct 20;2(4):765–73. doi: 10.1002/jha2.284 (PMC9175982; doi:10.1002/jha2.284)
Supplement: Supplementary file 1 — Table S1 [file JHA2-2-765-s001.docx]

**Supplementary Table 1. Result of univariate Cox regression analysis for overall survival and progression-free survival**

| Variable | Category | Overall survival | | | Progression-free survival | | |
| --- | --- | --- | --- | --- | --- | --- | --- |
|  |  | Hazard ratio | 95% CI | p-value | Hazard ratio | 95% CI | p-value |
| Age (y/o) | ≥60 | 1.36 | 0.71-2.63 | 0.358 | 0.98 | 0.64-1.50 | 0.928 |
| Gender | Men | 1.74 | 0.87-3.48 | 0.119 | 1.26 | 0.82-1.92 | 0.294 |
| ISS | ≥2 | 1.12 | 0.58-2.17 | 0.437 | 1.46 | 0.95-2.55 | 0.083 |
| PS | ≥2 | 0.37 | 0.11-1.23 | 0.066 | 0.96 | 0.54-1.71 | 0.890 |
| M protein | IgG | 0.80 | 0.42-1.55 | 0.511 | 0.90 | 0.59-1.39 | 0.643 |
| Maintenance | + | 0.76 | 0.39-1.49 | 0.427 | 0.69 | 0.45-1.06 | 0.090 |

ISS, International Staging System; PS, performance status; CI, confidence interval.
